# Comparison between two Canadian Provinces on technology use for social interaction by older adults: comparative cross-sectional survey study

**DOI:** 10.1186/s12877-025-06133-y

**Published:** 2025-07-03

**Authors:** Kayoung Lee, Theodore D. Cosco, Shelley Peacock, Megan E. O’Connell, Kristen R. Haase

**Affiliations:** 1 School of Nursing, Faculty of Applied Science, University of British Columbia, T201-2211 Wesbrook Mall, Vancouver, BC V6T 2B5 Canada; 2 Department of Gerontology, Simon Fraser University, Vancouver, BC Canada; 3 Oxford Institute of Population Ageing, University of Oxford, Oxford, UK; 4 School of Public Policy, Simon Fraser University, Vancouver, BC Canada; 5 College of Nursing, University of Saskatchewan, Saskatoon, SK Canada; 6 Psychology & Health Studies, University of Saskatchewan, Saskatoon, SK Canada

**Keywords:** Older Adults, Technology use, Social interaction, COVID-19

## Abstract

**Background:**

The COVID-19 pandemic prompted most people to embrace different approaches to their daily activities. Due to various measures to slow transmission in many jurisdictions, older adults were particularly impacted by measures restricting interactions. Our previous cross-sectional study identified barriers and facilitators for technology use for social interaction among older adults in British Columbia (BC), Canada. To investigate whether regional differences exist, the same survey from the previous study was conducted in Saskatchewan (SK), Canada during the same time. We also explored whether education and income levels were associated with older adults’ social technology usage.

**Methods:**

The cross-sectional survey was conducted through random-digit dialing to older adults who were 65 or older in BC and SK. Data were analyzed through the Statistical Package for the Social Sciences (SPSS) software (IBM corporation) and Microsoft Excel. Thematic analysis was performed on the survey's responses to open-ended questions.

**Results:**

There were 806 participants, 400 from BC and 406 from SK. Education levels were associated with awareness of using technology for social interaction for both BC and SK while only SK had an association between new technology use and education levels. Similarly, income levels were also associated with awareness of technology use for social interaction for both provinces while only BC had an association between income levels to uptake of new technology. From the previous and current study, the barriers identified for technology use for social interactions in BC and SK were lack of interest, access (including financial issues) and physical limitations. SK participants identified perceived low self-efficacy as an additional barrier. For facilitators, BC and SK participants identified current and previous technology knowledge, help from others and motivation to keep social connections. Access to technology was unique to BC while better technology was unique to SK.

**Conclusions:**

Our study suggests that when older adults have access to resources to support their technology use, they will use them more, possibly enhancing their capacity for technology use. Future studies with more diverse populations around Canada may identify varying factors for older adults’ technology use and regional variations in how technology is used for social interaction.

**Supplementary Information:**

The online version contains supplementary material available at 10.1186/s12877-025-06133-y.

## Background

The COVID-19 pandemic created adjustments to how most people live, connect, and relate with one another. Governments implemented measures that prevented people from gathering, such as stay-at-home orders and physical distancing [1], and use of technology(ies) became common because limiting physical contact was the primary means of mitigating spread [2, 3]. This restriction, often mislabeled as social distancing (when physical distancing was the purpose), restricted social interaction. These isolation orders often affected older adults negatively in both their physical and mental health [4, 5]. Mortality and severity of COVID-19 also increased with age [6].

Our previous cross-sectional study identified several barriers and facilitators for older adults using technology for social interaction during the pandemic in British Columbia (BC), and Saskatchewan (SK), Canada ([7], O’Connell ME, unpublished data, 2024). During this time, we sought to explore whether there were regional differences in older adults using technology for social interaction during the pandemic. Relative to BC, SK, another Canadian province, has a smaller more rural population relative to the geography with ~ 30% of the population living in rural areas compared to ~ 10% in BC [8]. Because of a higher percentage of the rural population in SK, there may be more prominent effect of the digital divide, illustrating the inequity of technology access between rural and urban regions [9, 10]. BC also has 6 health authorities compared to 1 in SK, implying different measures taken for various visiting or socialization policies during the pandemic [11, 12].

 We hypothesized there may be a relationship between technology use for social interaction and education and income levels, and we hypothesized BC may show a more noticeable relationship than SK due to population differences. Based on a previous study [7], one of the barriers to technology use was a financial limitation, and there are also differences in reported incomes between BC and SK. According to Statistics Canada, in 2021, of 4,277,000 who reported income in BC, the average income was $58,400 and the median was $44,100 while of 865,000 who reported income in SK, the average income was $55,600 and the median was $45,000 [13]. Thus, we postulated income levels might affect the uptake of new technology or present knowledge of technology use, but we were unsure if these relations would vary by region and explored data from BC and SK in the current study.

## Methods

### Recruitment

The cross-sectional survey was conducted with older adults 65 or older in January and February 2021. The Canadian Hub for Applied and Social Research (CHASR) conducted the survey by using random-digit dialing, which is a survey approach where random phone numbers in both BC and SK in Canada are dialed.

### Data collection

Informed consent, age and residential status within the provinces were verified before asking demographics questions. As with the previous studies ([7], O’Connell ME, unpublished data, 2024), data on gender, education, and annual income levels were collected. The survey questions tried to capture awareness of and adaptation to new technology for social interaction, and barriers and facilitators associated with technology use in the older adult population of those who are ≥ 65 and older. The same questions were asked as in the previous studies across each province (Table 1) [7].

**Table 1:** List of survey questions

1. Are you aware of how you can use technology to connect with others? (Yes or No)
2. Since the beginning of the pandemic, are you using technology differently to connect with others? (Yes or No)
3. Was this new technology or had you used it before? (New technology or Used before)
4. Are you still using technology or did you stop? (Still using or Stopped)
5. What stops you from using technology to connect with others?
6. What would help or has helped you use technology to connect with others?
7. Are finances a barrier to your use of technology? (Yes or No)

### Data analysis

Data analyses were performed using Microsoft Excel and IBM SPSS Statistics (Version 29). Means and frequencies were measured for the characterization of the sample while the chi-square test, Mann–Whitney U Test and point-biserial correlation were performed for statistical analyses. All following sample size estimation was conducted using G*Power version 3.1.9.7 [14, 15]. For the chi-square test of independence, a priori power analysis was conducted to estimate a sample size of 108 with a medium effect size of 0.3 [16], an α of 0.05, a power of 0.8 and degrees of freedom of 2. Chi-square tests were performed to test the association between education levels and income levels to technology usage and uptake. For chi-square tests, we followed the assumption that no more than 20% of each expected frequency is less than 5 [17]. Effect sizes are reported as Cramer’s V [17]. When the sample size was smaller than the estimated sample size, Fisher’s exact test was performed [18]. As the responses did not have a normal distribution, Mann–Whitney U Test was performed to test the association between the age with the survey questions. Point-biserial correlation was performed to test the relationship between age and questions with dichotomous answers regarding technology use for social connection (i.e., Survey questions 1, 2, 3).

### Thematic analysis

The survey also included open-ended questions (i.e., Questions 5 and 6), one that asked about the barriers and facilitators for technology use. Each open-text answer was summarized as simple codes, codes were then organized into common themes as described by Braun and Clarke (2006) [19]. Responses were read, coded, and then reread to ensure coding accuracy. Two authors engaged in the coding structure with one author (KL) conducting the codes, and a second author reviewing and refining the codes (KRH). Two authors (KL and KRH) generated and agreed on the common themes for each barrier and facilitator open-text answer.

## Results

### Characterization of the sample

A total of 806 participants were recruited from BC (*n* = 400) and SK (*n* = 406). Participant ages ranged from 65 to 107 (M = 76.2, SD = 7.42). The demographics of participants from BC and SK, which include gender, education, and income levels are illustrated in (Table 2). From those who did not provide their income levels in BC and SK (i.e., those who answered don’t know or refused to answer), 174 out of 340 (51.2%) who reported education had high school as their highest level of education, 146 out of 340 (42.9%) had completed Bachelor’s degree as the highest education and 20 out of 340 (5.9%) had post university degrees such as Master’s, Professional Degree or Doctorate (Supplementary Table S1).

**Table 2:** Demographics of participants from BC and SK

	**BC**		**SK**		**Total**	
	**n**	**%**	**n**	**%**	**n**	**%**
**Gender**						
Male	145	36.3	158	38.9	303	37.6
Female	255	63.8	248	61.1	503	62.4
Total	400	100.0	406	100.0	806	100.0
Education levels^a^						
High school	135	33.8	180	44.9	315	39.4
University	201	50.4	198	49.4	399	49.9
Post university	63	15.8	23	5.7	86	10.8
Total	399	100.0	401	100.0	800	100.0
Income levels^b^						
< $25,000	41	16.5	34	15.9	75	16.2
$25,000 to $75,000	119	48.0	115	53.7	234	50.6
> $75,000	88	35.5	65	30.4	153	33.1
Total	248	100.0	214	100.0	462	100.0

^a^Education levels denote the reported highest level of education completed by the participants where post-university level includes: Master’s, Professional degree or Doctorate degree

^b^The income levels were divided to be consistent with the previously published paper., Haase et al. (2021)

### Relationship between education and technology use

As shown in (Table 3), the awareness of using technology for social interaction was associated with education levels, and there did not appear to be any regional differences in educational levels in BC and SK participants. The association strength as shown in effect size ranged from weak to moderate. Regional differences were apparent for new technological usage (Table 4). For BC participants, there was no association between education level and new use of technology. However, for SK participants, education level was associated with using new technology, and those with higher education adopted new technology more than less highly educated participants (Table 4). When total participants were tested, there was an association between education levels and the taking up of new technology.

**Table 3:** Chi-square test between education levels and awareness of technology use for social connection

	**Are you aware of how you use technology to connect with others?**								
	**Yes**		**No**		**Total**				
	**n**	**%**	**n**	**%**	**n**	**%**	***χ***^**2**^**(df**^**a**^**,n)**	**p**	**Effect size**
Education levels (BC)^b^									
High school	113	31.7	22	52.4	135	33.8	χ^2^(2,399) = 7.91	.019	.141
University	184	51.5	17	40.5	201	50.4			
Post university	60	16.8	3	7.1	63	15.8			
Total	357	100.0	42	100.0	399	100.0			
Education levels (SK)									
High school	141	40.6	37	71.2	178	44.6	χ^2^(2,399) = 18.09	<.001	.213
University	183	52.7	15	28.8	198	49.6			
Post university	23	6.6	0	0.0	23	5.8			
Total	347	100.0	52	100.0	399	100.0			
Education levels (BC, SK)									
High school	254	36.1	59	62.8	313	39.2	χ^2^(2,798) = 26.17	<.001	.181
University	367	52.1	32	34.0	399	50.0			
Post university	83	11.8	3	3.2	86	10.8			
Total	704	100.0	94	100.0	798	100.0			

^a^Degrees of freedom

^b^Education levels denote the reported highest level of education completed by the participants

**Table 4:** Chi-square test between education levels and technology adoption

	**Was this new technology or had you used it before?**								
	**New technology**		**Used before**		**Total**				
	**n**	**%**	**n**	**%**	**n**	**%**	***χ***^**2**^**(df**^**a**^**,n)**	**p**	**Effect size**
**Education levels (BC)**^**b**^									
High school	26	22.8%	31	34.8%	57	28.1%	χ^2^(2,203) = 5.07	.079	.158
University	60	52.6%	45	50.6%	105	51.7%			
Post university	28	24.6%	13	14.6%	41	20.2%			
Total	114	100.0%	89	100.0%	203	100.0%			
Education levels (SK)									
High school	27	34.6%	35	41.7%	62	38.3%	χ^2^(2,162) = 6.56	.038	.201
University	41	52.6%	47	56.0%	88	54.3%			
Post university	10	12.8%	2	2.4%	12	7.4%			
Total	78	100.0%	84	100.0%	162	100.0%			
Education levels (BC, SK)									
High school	53	27.6%	66	38.2%	119	32.6%	χ^2^(2,365) = 10.86	.004	.173
University	101	52.6%	92	53.2%	193	52.9%			
Post university	38	19.8%	15	8.7%	53	14.5%			
Total	192	100.0%	173	100.0%	365	100.0%			

^a^Degrees of freedom

^b^Education levels denote the reported highest level of education completed by the participants

### Relationship between income levels and technology use

When the chi-square test was performed to test the association between income levels and technology usage for social connection, an association was found for BC, SK and total participants as shown in (Table 5). The association strength was weak to moderate across each province based on effect size. It was inconclusive whether the new technology uptake was related to income levels. For the total number of participants, the income level was independent of technology adoption (Table 6). This was consistent in SK participants but for BC participants, the income level was associated with the new technology adoption. The association strength was moderate for BC. Since there were fewer than 108 in the sample size for SK participants who provided income levels and whether the technology was new or not, Fisher’s exact test was used instead of a chi-square test.

**Table 5:** Chi-square test between income levels and technology awareness for social connection

	**Are you aware of how you use technology to connect with others?**								
	**Yes**		**No**		**Total**				
	**n**	**%**	**n**	**%**	**n**	**%**	***χ***^**2**^**(df**^**a**^**,n)**	**p**	**Effect size**
Income levels (BC)									
< $25,000	35	15.6	6	26.1	41	16.5	χ^2^(2,248) = 8.10	.017	.181
$25,000 to $75,000	104	46.2	15	65.2	119	48.0			
> $75,000	86	38.2	2	8.7	88	35.5			
Total	225	100.0	23	100.0	248	100.0			
Income levels (SK)									
< $25,000	26	13.6	7	31.8	33	15.5	χ^2^(2,213) = 10.07	.007	.217
$25,000 to $75,000	101	52.9	14	63.6	115	54.0			
> $75,000	64	33.5	1	4.5	65	30.5			
Total	191	100.0	22	100.0	213	100.0			
Income levels (BC, SK)									
< $25,000	61	14.7	13	28.9	74	16.1	χ^2^(2,461) = 17.53	<.001	.195
$25,000 to $75,000	205	49.3	29	64.4	234	50.8			
> $75,000	150	36.1	3	6.7	153	33.2			
Total	416	100.0	45	100.0	461	100.0			

^a^Degrees of freedom

**Table 6:** Chi-square test between income levels and technology adoption

	**Was this new technology or had you used it before?**								
	**New technology**		**Used before**		**Total**				
	**n**	**%**	**n**	**%**	**n**	**%**	***χ***^**2**^**(df**^**a**^**,n)**	**p**	**Effect size**
Income levels (BC)									
< $25,000	9	11.4	10	16.4	19	13.6	χ^2^(2,140) = 7.08	.029	.225
$25,000 to $75,000	29	36.7	33	54.1	62	44.3			
> $75,000	41	51.9	18	29.5	59	42.1			
Total	79	100.0	61	100.0	140	100.0			
Income levels (SK)									
< $25,000	3	6.1	7	17.5	10	11.2	Fisher’s Exact test = 3.08^b^	.215	.189
$25,000 to $75,000	28	57.1	18	45.0	46	51.7			
> $75,000	18	36.7	15	37.5	33	37.1			
Total	49	100.0	40	100.0	89	100.0			
Income levels (BC, SK)									
< $25,000	12	9.4	17	16.8	29	12.7	χ^2^(2,229) = 5.44	.066	.154
$25,000 to $75,000	57	44.5	51	50.5	108	47.2			
> $75,000	59	46.1	33	32.7	92	40.2			
Total	128	100.0	101	100.0	229	100.0			

^a^Degrees of freedom

^b^Fisher’s Exact test was used for comparing income levels and technology adoption from SK participants as the sample size was less than 108 as calculated above

### Relationship between age and technology use

As shown in (Table 7), there was a significantly higher median age for participants who did not have an awareness of using technology for social connection compared to those who did. There was a small positive correlation between age and awareness of technology use for social connection. Similarly, there was a significantly higher median age for participants who used technology the same as before the pandemic compared to those who were using it differently, but the difference was only 1 year. There was a small positive correlation between age and differences in technology usage for social connection. There was no difference in the median age whether the technology was new or not; no correlation was found between age and technology uptake.

**Table 7:** Mann–Whitney U test and point-biserial correlation to test the relationship between age and technology use

		**Participants’ age (BC, SK)**							
		**median**	**n**	**U**	**z**	**p**	**r**	**r**_**pb**_^**a**^	**p**
Aware of technology									
Yes		74	699	44,965	6.43	<.001	.23	.25	<.001
No		82	91						
Use technology differently									
Yes		74	359	68,888	3.17	.002	.12	.11	.003
No		75	337						
New or used technology									
New technology		73	190	18,036	1.50	.133	.08	.08	.109
Used before		74	174						

^a^Point-biserial correlation coefficient

### Comparison of barriers to technology use for social connection between BC and SK

From the SK respondents, 53 out of 406 (13.1%) respondents reported not being aware of using technology for social interaction. Participants were further asked what the barriers were that stopped them from using technology for social interaction. The open responses were summed up into simple codes and then into themes. We organized these into three themes 1) low self-efficacy, 2) lack of interest and 3) internal and external factors including lack of access and physical and financial limitations. In comparison, the previous study reported the following factors as barriers to technology use for connection in BC: 1) lack of access, including financial issues; 2) lack of interest and 3) physical limitations. The differences and similarities between responses identified in BC and SK are illustrated in (Table 8).

**Table 8:** Barriers and facilitators affecting technology use for social connection in BC vs. SK

Barriers		Facilitators	
**BC**^**a**^	**SK**	**BC**^**a**^	**SK**
**Identified themes**			
Lack of access (includes financial)	Low self-efficacy	Technology knowledge	Technology knowledge
Lack of interest	Lack of interest	Help from others	Help from others
Physical limitations	Access, financial and physical limitations	Social motivation	Social connection
		Access to technology	Better technology

^a^BC factors are directly adapted from Haase et al., (2021)

The first identified barrier involved perceived low self-efficacy. Low self-efficacy includes participants who reported feeling unconfident in technology because of age or competency. This is a new theme that was not found in barriers from BC participants. For 29/37 participants whose answers involved low self-efficacy as a barrier to learning technology had high school as the highest completed education level. Many of the responses involved self-directed ageism and described that learning how to use technology itself was complicated. The following responses describe how the participant considered their age as a barrier to technology use. “*I guess I just consider myself too old*”. Another participant stated: “*I don’t have nothing. It’s the age*”.

Another prominent theme was the general lack of interest in technology use. The participants reporting these responses did not feel the need to use the technology or they were already satisfied with other means of connecting mediums such as telephone: “*I just never been interested in it[,] I guess. Never felt it was needed I guess.*”. Another participant reported: “*I just connect with others through the telephone.*”

Additional factors included lack of access, financial and physical limitations and mistrust of data privacy.

### Comparison of facilitators to technology use for social connection between BC and SK

In SK, 358 out of 406 people (88.2%) responded with facilitators that helped them use technology for social interaction. We also identified four main themes from SK participants 1) knowledge of using technology (including self-learning), 2) help from others, 3) keeping social connection with others and 4) better technology. The facilitators were mostly similar between BC and SK. Three out of four facilitators were congruent to each other. The difference was while BC participants had access to technology as one of the facilitators, this was not identified among SK participants.

The first identified facilitator involved having knowledge on using technology. As a part of technology knowledge, one of the subthemes involved self-learning where respondents answered they learned the technology themselves, saying: “*I just look stuff up on the internet*” and “*Just gradually learning how to do it I guess*”.

Another main theme as the facilitator for technology use was the help or learning from others, either family or friends. The respondents felt that help from others was the facilitator for using technology, reporting things like: “*Advice from our children*” and “*Available software on the internet*”.

The third facilitator included social pressure to use technology as found in a previous study for BC [7]. In SK, the participants also demonstrated the need to keep social relationship through the use of technology. For example: “*Oh[,] just the opportunity to keep in touch*” and “*Mostly I’ve been using the computer to contact others*”.

The last factor identified by SK participants was better technology where some participants identified speed as factor such as “*Higher internet speed*” or “*Faster internet speed*”. Another technological aspect was better systems such as “*I guess the advances in technology has helped. Smarter phones*” and “*I guess up-to-date phones*”.

## Discussion

In this paper, we conducted a comparative analysis of the changes in technology use for social interaction between two Canadian provinces. Our main finding is that the use of technology for social connection was associated with both education and income levels while the association between technology adoption to education and income levels differed between provinces. For SK participants, education played as an important factor in undertaking technology. BC participants’ education levels are more evenly scattered than SK participants’ education levels as per (Table 4). The BC sample was more highly educated compared to SK. Thus, this might have affected the differences shown in statistical analyses between the two provinces. As shown in (Table 5), income levels were associated with technology use for social connection. From the previous study in the United States, both education and income levels were associated with older adults’ online usage [20]. This suggests continued support may need to be incorporated to assist older adults with lower income in using technology as suggested previously, including using technology for social connection [21]. Lastly, (Table 7) shows that participants’ median age was higher for those who did not know how to use technology and slightly higher median age for those who have been using technology the same for social connection. This suggests higher age may be a factor in using technology for social connection and may affect trying new activities through technology.

With the increasing older adult population, it is crucial to involve older adults with technology [22]. In the study by Aleti and the colleagues from Australia, the authors identified that for older adults to progress through technology, they needed to learn by themselves, with others and by having someone to do the task for them [22]. The finding from this study shows the similarity to our study’s findings on facilitators of using technology for the connection. Our study also identified both self-learning and help from others, specifically with those in older adults’ social proximity. While having someone to manipulate the technology for an older adult was not found as a facilitator in our study, the family members and friends who are within social proximity of older adults were mentioned as those who help connect with technology.

Interestingly, the main themes for barriers to technology use for social interaction were slightly different between BC and SK. Lack of interest was a common theme found among older adults from both provinces as a barrier to technology. While lack of access was a common barrier in both BC and SK, this theme was less prominent compared to low self-efficacy and lack of interest among SK respondents. In contrast, for SK participants, one of the most prominent themes involved low self-efficacy, which participants related to their age, confidence, or proficiency. Education levels have been reported to affect the stance towards technology use among older adults- higher education levels showing a more positive perspective towards technology use [23]. This may imply that approaches to technology-related skills, for example, task lists (i.e., step-by-step guides on certain technology-related actions), can support older adults’ learning and technology uptake [24]. Self-efficacy, physical limitations, and perceived age limitations have been previously reported as barriers to using technology among older people [25]. Additionally, computer use and income levels (among other factors that were reported), were shown to significantly affect self-efficacy in older adults’ computer use [26]. On a positive note, the previous pilot study showed older adults can be educated to increase computer self-efficacy levels [27]. Thus, when older adults are supported to increase their self-efficacy with technology, their technology use may increase, which may include engaging in other technology-related activities [24]. In addition, because of the higher rural population in SK compared to BC, SK's older adult population may experience a digital divide more frequently [10]. Due to the digital divide, the population in rural areas experiences less technological-related access and information [10]. Therefore, the digital divide within rural populations in SK may have impacted participants from SK to feel less confident than their BC counterparts, possibly reporting low self-efficacy. Furthermore, varying exposure to technology as well as limitations in our survey questions about technology exposure could explain regional differences.

One notable difference between BC and SK participants is that BC participants felt that access to technology was a facilitator while SK participants reported better technology as one of the facilitators. This difference might account for the fact that SK has less population density than BC, 2.0 per km^2^ compared to 5.4 per km^2^ of BC [28], thus SK may not have better access infrastructure for IT, as low population density was reported as one of the reasons for Canada’s digital divide in rural regions [9]. Also, since the SK population is more sparse, there may not be as many locations and/or programs to access as BC participants. When considering the public health isolation policies between BC and SK during the pandemic, there does not seem to be a substantial difference between the two provinces [29, 30]. Both provinces applied social distancing and both had gathering restrictions while the number of restricted gatherings differed depending on the timeline-therefore this is not a substantial difference.

Based on the facilitators and barriers we found in our study, there could be several strategies to involve older adults in using technology, specifically for the purpose of using it to connect with others. Numerous studies previously reported associated factors related to technology adoption for older adults and the technology acceptance model for older adults such as age related to interest in computers, age and anxiety about computers, financial barriers, technological literacy, perceived value and emotions [31–34]. However, the pandemic changed perceptions and increased the necessity for frequency of use of technology among older adults, where some older adults have presented the pandemic as a favorable situation for utilizing technology [35]. These findings suggest that for older adults, while low self-efficacy may be a barrier to using technology, external circumstances and sufficient motivation can lead them to find technology favorable and adapt to the situation. For example, older adults who live alone indicate a more favorable stance toward technology acceptance [34] and positive perspectives were shared by older adults on technology use for healthy aging [36]. When the older adults regarded technology as simpler and more useful, they were inclined to accept and use technology services such as telemedicine [34, 37]. Based on the current literature and our study, it would be important to consider various aspects for older adults’ technology use and acceptance for socialization.

## Limitations

The participants recruited from both BC and SK are not representative of residents in each province. For example, the gender was determined based on the respondent’s voice and based on this information, our samples were characterized as 60% female and 40% male. This approach also would have missed those who do not identify as either male or female. As the participants were not asked what their self-identified gender was, our reported representation of gender as binary may be inaccurate and non-representative, as non-binary individuals were not represented. Additionally, many participants either refused or did not know about their income levels. This led to a smaller sample size for statistical analysis and for one test, having to use Fisher’s exact test instead of a chi-square test due to not meeting the calculated minimum sample size. We also acknowledge that while the survey questions were reviewed by the experts in the field, and the consistency of themes found from our current study show relevance, the formal validity and reliability test was not conducted.

Due to the nature of the telephone survey (random digit dialing), only limited demographic information was collected while the participants were randomly selected. For example, the demographic questions did not ask whether the participants lived in rural or urban areas. Thus, the assumption that the SK participants may live more in rural areas compared to the BC participants may not always be true. Thus, it should be taken with caution that by no means our study is the generalization of both provinces, even more so in Canada. As our study participants were limited to two provinces in Canada (i.e., BC and SK) due to resource and provincial-level research infrastructure limitations, future research could include participants from across Canada. In addition, because our study was conducted in only two Canadian provinces, we are unable to make a general statement regarding rural populations in other Canadian regions. In terms of technology adoption, only the income of BC participants was associated with technology adoption. This may speak to a sample that is not representative. Also, as a secondary analysis, some analyses done may be underpowered and subject to possible Type II errors. In addition, the survey questions asked about technology usage but the survey was conducted by telephone. This might suggest the participants may have more positive feelings towards technology. It would be beneficial to study whether each province has different results in the relationship between income levels and technology adoption for future studies. However, this study has strengths, including the large sample size, the qualitative and quantitative data, and the comparison of populations in two distinct regions.

## Conclusions

From our study, we conclude that BC and SK may share certain barriers and facilitators for technology use and also experience some distinct variances between provinces. The results suggest regional variations in the necessary resources to encourage, motivate or mitigate barriers to older adults'technology. Our findings suggest that when resources such as education or accessibility, become available, older adults’ technology use could be enhanced. Greater access to, and support to use technology(ies) could benefit older adults in diverse areas, including health management. Future research might aim to include a more diverse population from across Canada, not limited to two provinces. Also, it would be worthwhile to investigate the possible differences between western and eastern Canada with the cities that are comparable in population sparsity.

## Supplementary Information

Supplementary Material 1.

## Data Availability

Data are not publicly available as the participants of this study did not give written consent for their data to be shared publicly, therefore due to the sensitive nature of the research, supporting data are not available.

## Abbreviations

BC: British Columbia
SK: Saskatchewan
