# Supplementary material for: Comparison between two Canadian Provinces on technology use for social interaction by older adults: comparative cross-sectional survey study
Source: BMC Geriatr. 2025 Jul 3;25:489. doi: 10.1186/s12877-025-06133-y (PMC12224403; doi:10.1186/s12877-025-06133-y)
Supplement: Supplementary file 1 — Supplementary Material 1. [file 12877_2025_6133_MOESM1_ESM.docx]

Supplementary Table S1. Education levels of participants who did not report their income levels.

|  | | **BC** | | **SK** | | **Total** | |
| --- | --- | --- | --- | --- | --- | --- | --- |
|  | | **n** | **%** | **n** | **%** | **n** | **%** |
|  | |  |  |  |  |  |  |
| **Education levels^a^** | |  |  |  |  |  |  |
|  | High school | 73 | 48.0 | 101 | 53.7 | 174 | 51.2 |
|  | University | 64 | 42.1 | 82 | 43.6 | 146 | 42.9 |
|  | Post university | 15 | 9.9 | 5 | 2.7 | 20 | 5.9 |
|  | Total | 152 | 100.0 | 188 | 100.0 | 340 | 100.0 |

^a^Education levels denote the reported highest level of education completed by the participants where post-university level includes: Master’s, Professional degree or Doctorate degree.
